# Nucleotide-time alignment for molecular recorders

**DOI:** 10.1371/journal.pcbi.1005483

**Published:** 2017-05-01

**Authors:** Thaddeus R. Cybulski, Edward S. Boyden, George M. Church, Keith E. J. Tyo, Konrad P. Kording

**Affiliations:** 1 Department of Physical Medicine and Rehabilitation, Rehabilitation Institute of Chicago, Northwestern University, Chicago, Illinois, United States of America; 2 Media Lab, Massachusetts Institute of Technology, Cambridge, Massachusetts, United States of America; 3 Department of Biological Engineering, Massachusetts Institute of Technology, Cambridge, Massachusetts, United States of America; 4 McGovern Institute, Massachusetts Institute of Technology, Cambridge, Massachusetts, United States of America; 5 Biophysics Program, Harvard University, Boston, Massachusetts, United States of America; 6 Wyss Institute, Harvard University, Boston, Massachusetts, United States of America; 7 Department of Genetics, Harvard Medical School, Harvard University, Boston, Massachusetts, United States of America; 8 Department of Chemical and Biological Engineering, Northwestern University, Evanston, Illinois, United States of America; 9 Department of Physiology, Northwestern University, Chicago, Illinois, United States of America; 10 Department of Applied Mathematics, Northwestern University, Evanston, Illinois, United States of America; Temple University, UNITED STATES

## Abstract

Using a DNA polymerase to record intracellular calcium levels has been proposed as a novel neural recording technique, promising massive-scale, single-cell resolution monitoring of large portions of the brain. This technique relies on local storage of neural activity in strands of DNA, followed by offline analysis of that DNA. In simple implementations of this scheme, the time when each nucleotide was written cannot be determined directly by *post-hoc* DNA sequencing; the timing data must be estimated instead. Here, we use a Dynamic Time Warping-based algorithm to perform this estimation, exploiting correlations between neural activity and observed experimental variables to translate DNA-based signals to an estimate of neural activity over time. This algorithm improves the parallelizability of traditional Dynamic Time Warping, allowing several-fold increases in computation speed. The algorithm also provides a solution to several critical problems with the molecular recording paradigm: determining recording start times and coping with DNA polymerase pausing. The algorithm can generally locate DNA-based records to within <10% of a recording window, allowing for the estimation of unobserved incorporation times and latent neural tunings. We apply our technique to an *in silico* motor control neuroscience experiment, using the algorithm to estimate both timings of DNA-based data and the directional tuning of motor cortical cells during a center-out reaching task. We also use this algorithm to explore the impact of polymerase characteristics on system performance, determining the precision of a molecular recorder as a function of its kinetic and error-generating properties. We find useful ranges of properties for DNA polymerase-based recorders, providing guidance for future protein engineering attempts. This work demonstrates a useful general extension to dynamic alignment algorithms, as well as direct applications of that extension toward the development of molecular recorders, providing a necessary stepping stone for future biological work.

## Author summary

This work demonstrates a necessary computational tool for the development and implementation of molecular recorders, a promising potential technique for massive-scale neuroscience. Molecular recorders use proteins to encode levels of a substance we want to measure (e.g. calcium in neural applications) as detectable changes in a linear cellular structure, e.g. misincorporations in a strand of DNA, or fluorescent proteins traveling down a microtubule. This encoding represents levels of the measured substance over time, much like a ticker tape represents information linearly on a strip of paper. The unique intracellular nature of this approach promises a significant scaling advantage over current techniques. The molecular recording approach suffers a particular drawback involving timing: unlike most methods of recording signals, in simple molecular recording systems we do not observe when each data point was recorded. This timing information is almost always required in order to make associations between our recorded data and the rest of the experiment. In this work, we propose a method to estimate the timing of these data points using easily-observable experimental measurements. We demonstrate the application of this method in a simulated neuroscience paradigm, investigate the effect of experimental design on this method, and determine protein properties that would be desirable in molecular recorders. These findings are useful both as a computational proof-of-concept, and as guidelines for current efforts to engineer proteins for molecular recording.

## Introduction

As we seek to understand complex questions in neuroscience, we are increasingly interested in the feasibility of massive-scale methods for neural recording [1–5]. One such proposed method is molecular recording, which uses engineered DNA polymerases (DNAPs) to encode information about neural activity onto a newly synthesized DNA strand, such that the position in the DNA sequence corresponds to the order and approximate timing of recorded events [6–8]. Rather than reading out neural activity from an electrode or photodiode during an experiment, molecular recorders would store neural activity intracellularly. This information would not be read out in real-time, but *post-hoc* using high-throughput DNA sequencing. The recording DNAPs could be genetically encoded and selectively expressed in neurons, allowing us to obtain activity records from large populations of cells. DNAP-based recording techniques promise an inherently ultrahigh-scale neural recording technique, building off of advances in biotechnology and computational power. However, significant hurdles remain in realizing such a technology.

While molecular recorders promise massive-scale neural recording, they do not inherently provide all the data obtained using current recording techniques. With current techniques, e.g. electrical or optical recording, data about the timing of each sample is recorded alongside the desired recording. With DNAP-based recorders, we sample data using DNA sequencing, which occurs after an experiment has concluded. That is, without any inherent clocking mechanisms, the output from molecular recorders lacks any explicit timing information about what it recorded. Without timing information, recorded neural activity cannot be interpreted in the context of other signals observed during experiments, e.g. movement or delivered stimulus. The central problem here is that we do not know which nucleotides were written at which times, i.e. we cannot link our representation of neural activity to things we observe in the outside world. Thus, the timing of data from molecular recorders must be inferred or estimated before it can be useful to understand the brain.

Due to the stochasticity inherent in DNAP activity (or that of any protein), it is difficult to predict when a nucleotide was incorporated *de novo*. Uncertainty in timing estimates result in uncertainty about the underlying signal; without timing information, signal estimates become highly inaccurate, providing at most a few seconds of reliable recording under common conditions [7]. However, if we observe experimental data that should be correlated with neural activity during our experiments, we can generate predictions of what possible patterns of neural activity we might observe given that data. This, in turn, can provide some information about the timing of nucleotide incorporations: if we see a particular pattern of activity in our DNA-based record, the DNA was likely written by a neuron whose tuning would generate a similar activity pattern in response to the experimental variables we observe, and at a time where the neuron would have generated that pattern. If we enumerate the ways in which we believe a neuron could respond to the observed experimental variables in question, we can search for the most-likely response given the DNA-based record we observe. It is worth stating that this type of approach is not model-free, and there are many situations where this assumption of a tuning model is inappropriate, i.e. in areas of the nervous system that we either model poorly or do not know what form a model would take. However, in areas where we have reliable modeling approaches or seek to evaluate particular models, a model-based approach may be able to provide considerable insight.

One way to utilize these models to estimate timing is the one we use here: generate predictions of neural activity with known timing using observed experimental variables, then find the globally most-similar alignment between those predictions and our recorded data. This class of alignment problems is frequently found in the time series analysis domain, e.g. in speech or signature recognition [9–11]. Dynamic time warping (DTW) is an efficient solution to this class of alignment problems, determining the optimal alignment between the template and signal using dynamic programming principles. With a probabilistic interpretation, DTW allows us to infer the most likely timing of a signal with respect to a given template, as well as determining the most likely template from a set of possible templates [12]. These properties make DTW-class algorithms uniquely suited for the determination of signal timings for molecular recorders.

Given that we are interested in applying this algorithm to massive-scale datasets, we are immediately interested in algorithms that can efficiently harness large-scale computing resources. As DTW is a dynamic algorithm, with successive steps depending on previous calculations, it is difficult to apply asynchronous computing approaches, at least on an algorithm level. Thus many, though not all, parallel approaches to DTW have largely focused on task-level parallelism rather than parallelizing cost computation [13–18]. As a result, for computationally-intensive individual alignments, it tends to be difficult to fully utilize the massively parallel computing resources that are becoming more common. A highly-parallelized dynamic alignment algorithm would be useful for a number of reasons.

Here we describe a parallelized step-pattern variant of DTW with applications to the analysis of molecular recorder output. We demonstrate the algorithm’s ability to accurately determine incorporation times for single DNA strands generated by a simulated molecular recorder, compensating for the timing issues inherent in protein-based molecular recorders. We demonstrate the utility of this algorithm in practice through simulated neuroscience applications, and use simple simulated experiments to explore how DNAP parameters such as speed and error rate affect the accuracy of our timing estimates. Through proposal and application of this algorithm, we present findings relevant for current biological research into molecular recording.

### Algorithm and experimental overview

Our algorithm solves a problem central to interpreting molecular recorder output in the context of neural recording: it aligns a single DNA-based record to an estimate of neural activity. We evaluate the local likelihood of each nucleotide being written at any time within some recording window given some assumed neural and DNAP properties. Then, using a dynamic programming-based technique, we attempt to find a global alignment given the local likelihoods and a prior defined by the DNAP kinetics. This algorithm is similar in structure to Dynamic Time Warping, utilizing a modified step pattern that reflects certain biological realities (See Algorithm Methods,S1 Fig). The step pattern limits the possible search space by enforcing these constraints: 1) nucleotides cannot be aligned to the same time point, 2) nucleotides can only be aligned to one time point, and 3) there can be a variable amount of time between incorporation of two adjacent nucleotides. We weight the potential options from this step pattern so that alignments made more likely by DNAP kinetics are favored. Notably, this approach enables significant algorithm parallelism, emerging from the constraint that nucleotides can only be aligned to one time point. As there are no dependencies between possible alignments of a given nucleotide, we can calculate the costs of all possible alignments of a given nucleotide concurrently.

**Fig 1:**
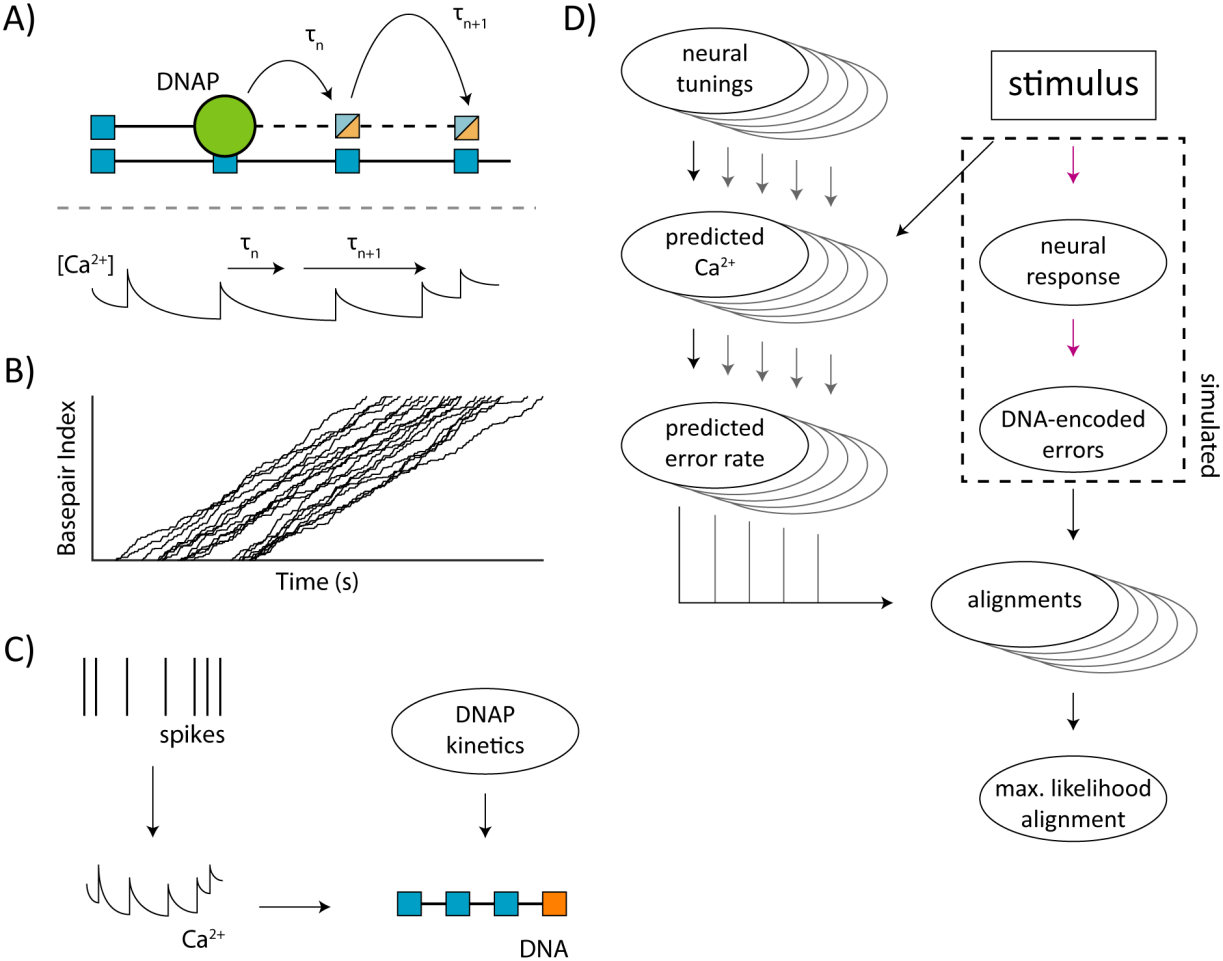
Procedural overview. A) Molecular recording overview. A DNAP (green) copies a template DNA strand of known sequence. It can incorporate the correct Watson-Crick paired nucleotide (blue) or make an error and incorporate a non-paired nucleotide (orange). These incorporations and misincorporations can be read out via DNA sequencing. The time τ between these nucleotide incorporations is variable and a function of DNAP kinetics. While these nucleotides have regular DNA-based indexing, they have irregular indexing with respect to time. B) Examples of nucleotide-time mappings, simulated as described in Methods. Stochastic DNAP kinetics produce non-linear nucleotide-time mappings. Further, diffusion and other biological processes can lead to non-uniform recording start times. C) Generative model for DNA-based error signals. Neural spikes lead to elevated calcium levels in the neuron. These changes in calcium alter the instantaneous error rate of a molecular recorder. These changes in error rate are only recorded when nucleotides are incorporated into a DNA strand, causing the resulting DNA-based record to be a function of cellular calcium and DNAP kinetics. D) Overview of alignment and inference. We begin with a set of potential neural tunings and a time-varying stimulus. The stimulus is transformed by the neuron to neural activity, which is then recorded as errors in a DNA strand by a molecular recorder. In parallel, we use the set of potential neural tunings in combination with the observed stimulus to generate estimates of neural calcium and the resulting instantaneous DNAP error rate. We use our algorithm to align the DNA-based errors to each of the estimated error-rate traces, then select the maximum-likelihood alignment. Dashed box indicate biological processes that are simulated in parts of these analyses.

**Fig 2:**
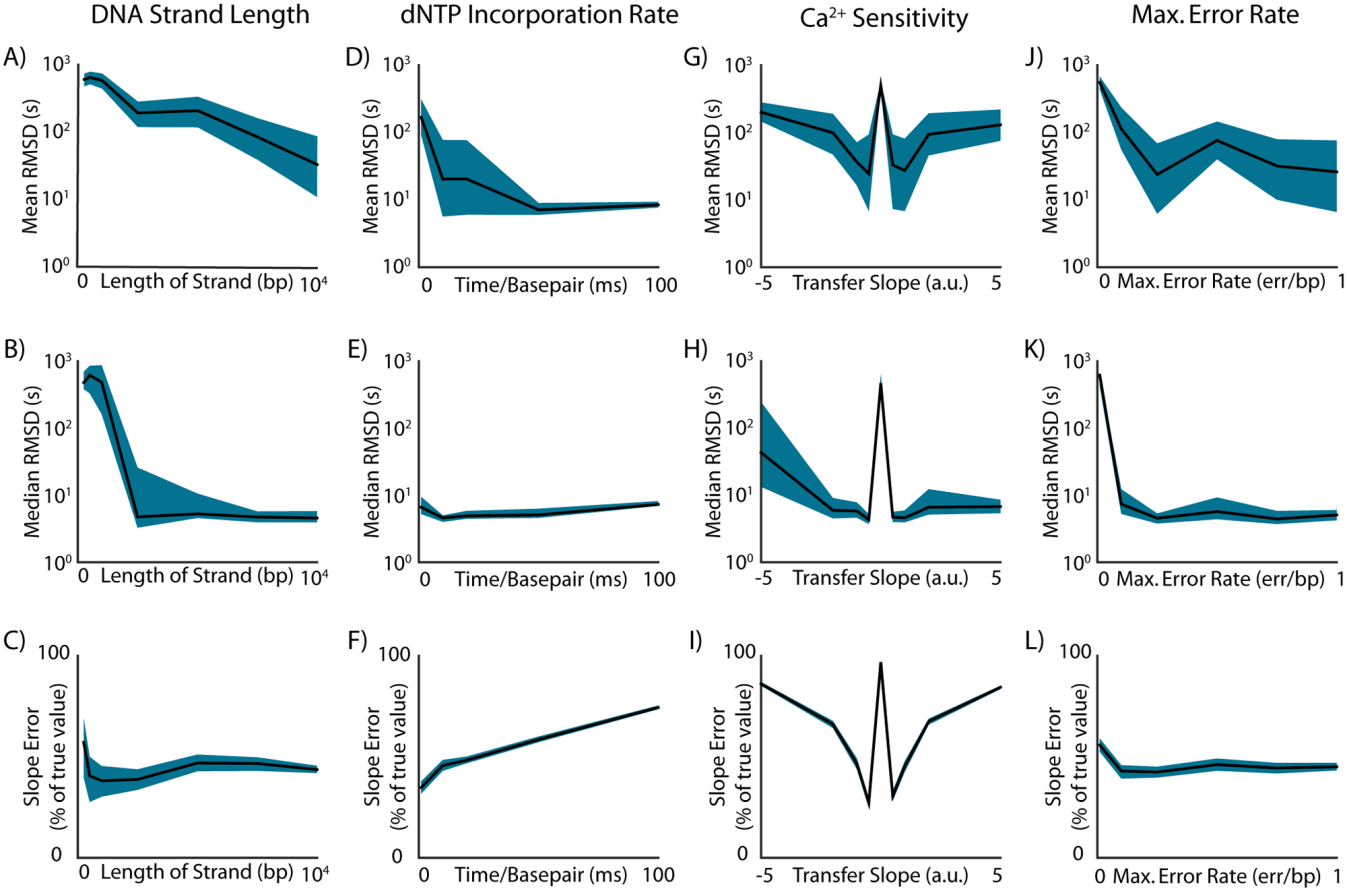
Effect of DNAP parameters on alignment and tuning estimation. Examining alignment performance using simulated DNAPs with varying parameters. Bootstrapped 95% confidence intervals of displayed values are indicated by blue silhouettes. **A,B)** The mean and median timing RMSD of alignments for DNA-based records of increasing length. **C)** Error in slope estimation for DNA-based records of increasing length. **D,E)** The mean and median timing RMSD of alignments for DNAPs with decreasing nucleotide incorporation rates. **F)** Error in slope estimation for DNAPs with decreasing nucleotide incorporation rates. **G,H)** Mean and median timing RMSD of alignments for DNAPs with increasing sensitivity to [Ca^2+^]. **I)** Error in slope estimation for DNAPs with increasing sensitivity to [Ca^2+^]. **J,K)** The mean and median timing RMSD of alignments for DNAPs of increasing maximum error rate. **L)** Error in slope estimation for DNAPs with increasing maximum error rate.

In order to demonstrate the utility of this algorithm, we apply our technique to simulated output of molecular recorders (Fig 1A), demonstrating various aspects of algorithm performance as well as exploring the ability of DNAPs to encode neural information. The general experimental pipeline consists of four parts: (1) simulation of a molecular recording experiment (Fig 1B and 1C), (2) alignment of single recorder outputs to a set of time-indexed expected DNAP error rates, which represent potential neural tunings to observed experimental covariates, (3) selection of a template that best matches the molecular recorder output (Fig 1D), and (4) inference of neural parameters using time-aligned DNA-based signals (see Methods).

We simulate a biologically-inspired generative model with several parts: (1) an explicit parameterized model of how neural activity either depends on a stimulus or results in observed behavior (Neural Tuning), (2) how this neural activity modulates DNAP error rate, via Ca^2+^ concentration or other mechanisms (DNAP Tuning), and (3) a probabilistic description of DNAP kinetic properties, e.g. incorporation rate and pausing (DNAP Kinetics). This generative model can be parametrized using existing knowledge about neural and polymerase properties where known. In this paper, we use DNAPs with optimistic DNAP error tuning, i.e. maximum error rates higher than many DNAPs with incorporation rates suitable for recording, but with otherwise-realistic properties [19–21]. We also assume knowledge of these system characteristics (apart from neural tuning) in order to parametrize the alignment algorithm.

Given simulated DNA output and a time-varying input to the system, we iterate over potential neural tunings to find a tuning that provides an alignment most consistent with the observed DNA-based signal. We then use this *maximum a posteriori* alignment to generate a time-indexed DNA signal, and use this signal to infer neural parameters. We evaluate algorithm performance both by accuracy of timing estimation, i.e. how many seconds estimated incorporation times differ from true incorporation times on average, and accuracy of inferred neural tuning parameters, i.e. how the estimated behavior of a neuron differs from the true neural behavior. Specifically, to evaluate accuracy of timing estimates, we examine the root-mean square deviation (RMSD) between the estimated timings and the true incorporation times for a given alignment.

There is a highly non-linear relationship between alignment “success” and timing accuracy, as nearby alignments do not necessarily have similar likelihoods. Thus, we provide both a mean and median value for timing accuracy when those values differ by a large amount. To evaluate tuning accuracy, we estimate tuning parameters from the aligned DNA data and examine the distance between the algorithm-estimated parameters and those derived directly from the recorded neural data, which we treat as ground-truth for these studies.

## Results

### Performance comparison to traditional DTW

Before exploring algorithm applications, it is worth exploring the performance implications of this approach. It bears mentioning again that, while they do not calculate the same cost function, our algorithm and traditional DTW are closely related; both are dynamic programming algorithms with effective worst-case complexity of *O*(*NT*) where N and T are the lengths of the two inputs being aligned. As we have mentioned, our algorithm has significant differences in implementation that allow it to be substantially parallelized; this allows for substantial performance increases using modern computing devices (See Algorithm Methods). While a naïve implementation of our algorithm performs more slowly than traditional DTW for a given set of inputs, parallelized implementations substantially outperform traditional DTW (S1 Fig). We observe up to a 16x speedup over traditional DTW when using a GPU-based implementation of our algorithm on a personal computer, and up to a 5x speedup when using a CPU-based implementation.

### Acceptable parameters for DNAP-based recorders

The feasibility of a “ticker tape” DNA-based recording scheme depends heavily on the properties of the DNAP used. For instance, the length of records (in base pairs) influences how much information is contained about neural activity, and thus impacts algorithm performance. Similarly, the speed, pausing, and fidelity properties of the DNAP used influence the information about neural activity contained in a DNA-based record [7]. Here, we look to determine the effect of these properties on the accuracy of our algorithm, and thus the expected performance of a molecular recording setup. Determining these effects allow us to form guidelines as to what kinds of DNAPs would be required for successful recording and alignment.

We use an entirely-simulated experiment here, i.e. we fully know the tuning linking stimulus to neural activity. This allows us to isolate the effects of DNAP properties on alignment from the effects of inaccurate neural activity estimates. We simulate a neuron with a linear response to an artificial stimulus; we deliver random levels of stimulus in 5s blocks over the course of 2000s (~30 minute recording window), and simulate the neuron’s spiking activity and intracellular calcium. We then simulate the output of a molecular recording system during that time period. We then align the molecular recorder output to the true stimulus signal. Using this simulation, we can focus on error induced by the DNAP and alignment algorithm in isolation.

We aim to estimate nucleotide incorporation timings, as well as the strength of the neuron’s tuning to the stimulus, i.e. the slope of the neuron’s tuning curve. The best alignments possible under this scheme have timing error up to the size of the stimulus features (5s); alignments with timing error less than this are generally considered to be accurate. Error with respect to tuning parameter is presented as a proportion of the true parameter. Except for the DNAP parameter being varied, the simulated DNAPs are identical (~100 Hz, mean pause duration of 2s; see Methods).

As record length increases, finding a randomly generated pattern that resembles the record becomes less likely, and alignment to a unique site should become easier. However, from a biological perspective, generating longer sequences may be more difficult, requiring polymerases with specialized properties, e.g. high processivity, high activity, or strand-displacement activity. Thus, it is useful to know minimal record lengths for successful alignment. When we increase record length in our simulations, we indeed find a resulting decreasing timing error. Generally, we find that records with length longer than 2.5K basepairs align with <5s median timing error (Fig 2A and 2B). Interestingly, we find that slope estimation is relatively constant regardless of record length, suggesting that, while record length is crucial to timing estimation, information about neural tuning in the record is not necessarily absent in shorter records (Fig 2C).

DNAP speed effectively changes the sampling rate of our system; if we have a slow DNAP, we can record for longer periods of time for a given strand length, but also record less information about any given interval. If we are interested in longer time-scale phenomena (e.g. environmental sensing, medical diagnostics) [22], we may wish to use slow DNAPs. However, due to the low sampling rate, we may not be able to recover useful information about timing and tuning in a neural paradigm. In our simulated stimulation paradigm, we find that slower DNAPs in fact increase timing accuracy (Fig 2D). However, median timing error stays relatively constant as speed decreases, implying that slow DNAPs simply decrease the amount of extreme timing errors we observe (Fig 2E). This runs parallel to our observations about record length; aligning to a longer time-indexed template is easier than aligning to a short one. However, our accuracy in determining tuning parameters decreases as we use slower DNAPs (Fig 2F). This indicates that we should, in general, be using fast DNAPs if we are interested in recovering tunings [19]. Meanwhile, slower DNAPs can provide longer records for a given strand length at the expense of diluting the information they carry about underlying phenomena.

Another property of DNAPs that can affect the quality of recordings is the transfer function relating analyte (e.g. calcium) concentration to error rate, *f*(·). We have modeled *f*(·) as a sigmoid with three parameters:

$$f\left(C\right)=R_{max}\cdot \frac{1}{1+\text{exp}\left[b\left(C-C_{0}\right)\right]}$$ (1.1)

where *C*_0_ denotes the [Ca^2+^] that leads to half-maximum error rate, *b* denotes the steepness of the response curve, and *R*_*max*_ denotes the maximum error rate of the DNAP. When selecting (or engineering) DNAPs to record with, we will need to optimize over these parameters. Here, we analyze DNAPs with varying transfer function slopes *b*, i.e. varying sensitivities to [Ca^2+^], ranging from step-like DNAPs to DNAPs with a wide dynamic range. We find that DNAPs with moderate sensitivities to [Ca^2+^] provide the most accurate timings, while both step-like and overly shallow transfer functions decrease alignment accuracy (Fig 2G and 2H). We find similar results for parameter estimation (Fig 2I), where appropriately-sloped DNAP tunings provide better estimates of neural parameters than DNAPs that are either too insensitive (low |*b*|) or too step-like (high |*b*|) with respect to [Ca^2+^]. This adds evidence to an assumption many investigating molecular recording techniques have been working under: DNAPs will have to be tailored in order to achieve optimal recording of even simple signals.

We are also interested in how the maximum error rate *R*_*max*_ affects alignment accuracy. This is of particular interest from a biological perspective: many natural DNAPs with incorporation rates suitable for high-resolution recording have low error rates. It is useful to understand what minimal error rates would be feasible for molecular recorders, as well as examine system performance as *R*_*max*_ scales. Here, we consider DNAPs that have near-zero error rates at low [Ca^2+^], and increase to some maximum error rate *R*_*max*_ under high [Ca^2+^] conditions. We find that alignment accuracy increases as maximum error rate increases (Fig 2J and 2K), as expected. Interestingly, we find that parameter estimation is relatively insensitive to *R*_*max*_. Again, this seems to suggest that while timing accuracy tends to degrade with unfavorable DNAP parameters, molecular recorder output tends to retain information about underlying neural tuning.

### Application to a center-out reaching task

Here, we demonstrate the feasibility of molecular recorders in a conventional neuroscience experimental paradigm. We analyze single-unit neural data recorded from M1 and pre-motor cortex during a center-out reaching task in a rhesus macaque, estimating the preferred movement directions of recorded neurons (data obtained from the DREAM reaching experiment database, see Flint 2012 for details [23–25]). We use the recorded spikes as the basis for simulated calcium transients and molecular recorder output. We also generate a set of estimates of neural activity from the kinematic data recorded during the task, with estimates representing velocity-tuned neurons with preferred directions distributed uniformly on [0, 2*π*]. Here, we use eight activity estimates as alignment templates. We apply our alignment algorithm to this data, aligning the molecular recorder output to each of the estimates, then selecting the maximum-likelihood alignment. The result, an estimated mapping of nucleotides to time, allows us to generate tuning curves for the recorded neurons. From this, we can estimate neural tuning parameters and infer how neural activity is modulated with respect to the recorded kinematics (details in Methods). The alignments here encounter alignment- and DNAP-based error, as in the previous section, but also encounter biology-based error when estimating neural activity from kinematic data. Thus, these experiments serve as an estimate of molecular recorder performance in a real-world scenario.

**Fig 3:**
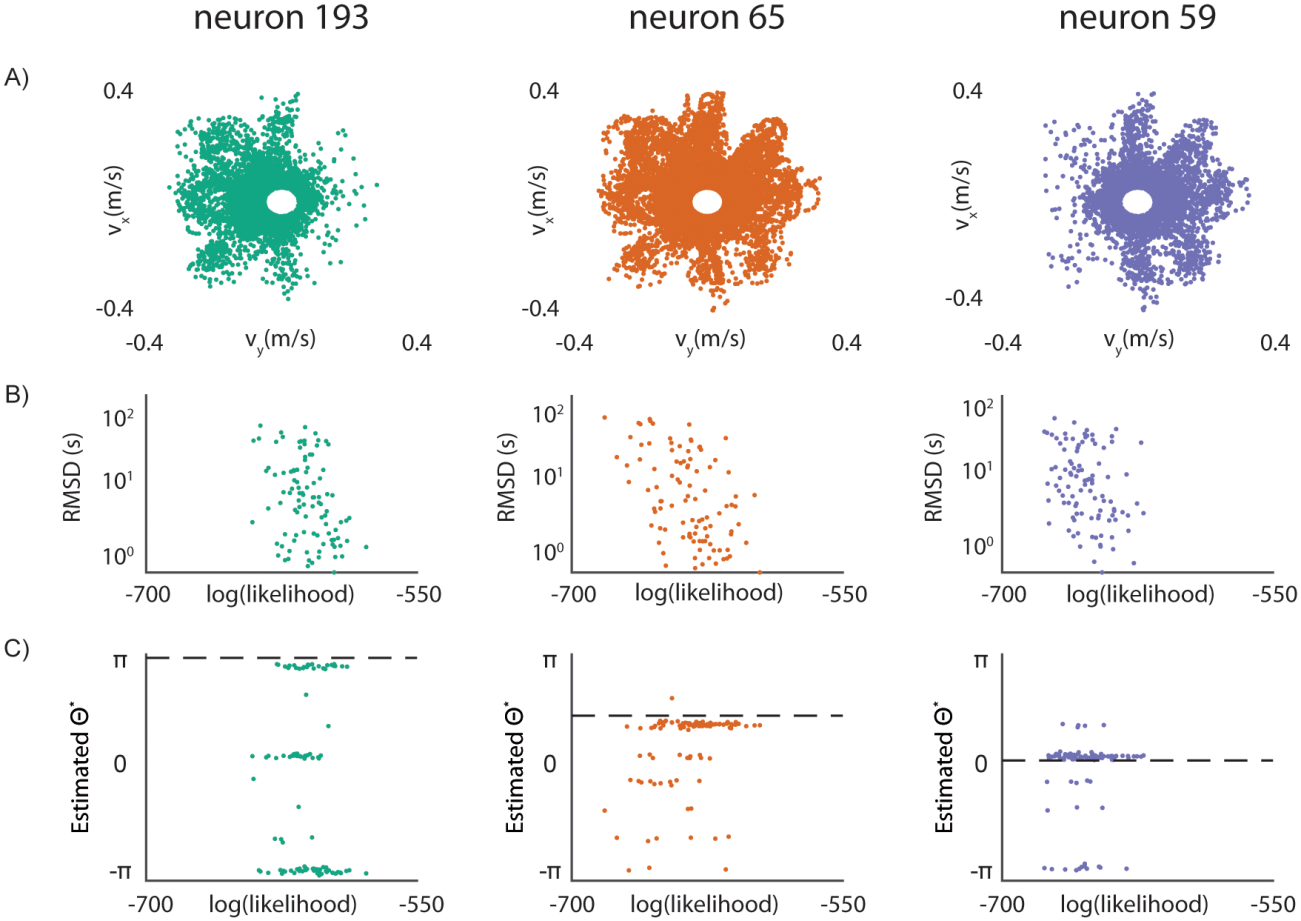
Determination of tuning parameters in neurons. Data for each of the three analyzed neurons are displayed as columns. **A)** Neural activity plotted as a function of cursor velocity in 3 selected neurons from the Flint 2012 dataset. Points represent neural spikes, locations indicate hand velocity during the spike time. **B)** Timing error (RMSD) as a function of alignment likelihood for model-derived timings in 3 selected neurons. Each point represents the most-likely alignment of the DNA-based record to one of eight activity estimates. Each point represents one of 100 trials. **C)** Estimated neural preferred direction as a function of alignment likelihood for the 3 selected neurons. Each point represents the preferred reach direction generated from the best alignment of the DNA-based record. Dashed lines indicate the preferred direction of the neuron, estimated from neural activity data. Each point represents one of 100 trials.

Using a plausible set of DNAP parameters (~100 Hz incorporation rate, mean pause duration of 2s, ~17% of time spent paused; see Methods for further details), we find that we are generally able to recover rough timing estimates and accurate tuning parameters from the simulated molecular recording experiment. As an initial demonstration, we examine several neurons that exhibit high firing rates and significant directional tuning (Fig 3A). Under these conditions, we are able to estimate nucleotide timings to within an average of ~15s (95% confidence intervals for average trial RMSD: [10.0,16.5], [12.1,20.3], and [14.8,22.5] seconds, Fig 3B). While timing accuracy is lower than desired, particularly for experiments that require sub-second precision using current techniques, these alignments still allow us to generate the estimated neural tuning direction θ* with error of ~10% (average errors of 0.5, 0.3, and 0.3 radians, Fig 3C). Median timings are substantially better than average timings across the board (95% confidence intervals for median trial RMSD: [3.8,7.2], [3.1,8.7], and [6.5,13.7] seconds).

Some of the error we encounter when generating alignment estimates may stem from our discrete parametrization of neural tunings. That is, we may not provide an estimate of neural activity similar enough to the true activity in order to generate accurate alignments. We can examine the contributions of this effect to algorithm accuracy by supplying a neural activity estimate generated using the neural tuning estimated from electrophysiology data, the best possible estimate we can provide given a particular model. Indeed, if we supply a neural activity estimate generated using the ground-truth neural preferred direction in our motor control experiment (rather than the 8 naïve preferred directions), we substantially reduce both timing error and error in θ* (S2 Fig). While we do not know the true preferred direction *a priori* and this kind of analysis could not be performed in practice, this suggests that a large portion of observed error can be attributed to the discrete parametrization of the search space. Increasing the resolution of the search space should improve alignment accuracy at the expense of execution time.

We apply our algorithm to each neuron in the dataset, examining aggregate performance over a population of recorded neurons. We find that the technique has middling performance on the whole dataset, only able to estimate timings to within 24s for 12% of neurons recorded (S3 Fig). If we limit the set of analyzed neurons to those that have substantial reach-modulated activity (model pseudo-R^2^ > 0.05, firing rate *λ* > 20 spikes/s), this improves to 47%. We are able to estimate preferred direction to within ±0.2π (±36°) for 39% of the dataset; this improves to 59% of the reach-modulated neurons (S3 Fig). While this filtering does not explain all observed error, it is useful when reconciling the results for individual neurons in Fig 3 with the larger dataset. This improvement upon filtering for active, well-modeled neurons demonstrates two things: 1) this method performs poorly on sparse-firing neurons, and 2) this method performs poorly on neurons that are not well-described by the set of models we consider. Both of these shortcomings are as expected given the algorithm. The former can be addressed by evaluating average neural activity represented by a DNA-based record, which can be done in a naïve, model-free manner. The latter, an inability to align signals that we cannot already model accurately, remains a shortcoming of this approach when attempting the interpretation of molecular recorder output.

We also analyze recording systems with a hypothetical DNAP that exhibits no pausing, but is otherwise identical to the previous DNAPs (see Methods). When examining the same neurons as above, we find drastically decreased timing errors (RMSD 95% CIs of [0.17,0.18], [0.31,0.39], and [0.47,3.0] seconds) and parameter estimation errors (average errors of 0.1, 0.2, and -0.04 radians, S4 Fig). Using these highly optimized DNAPs, we approach the timing resolution that would seem to be useful for high-precision neuroscience experiments, and retain high-accuracy prediction of neural tunings. A conclusion from this analysis is that much of the error we observe with our technique resolves when DNAPs behave more regularly. These results are of particular interest to us because of their biological implications: DNAP pausing generally has both DNAP-based and sequence-dependent components, and can be ablated using sequence context, chemical, or temperature-based means [19,26,27]. This significant improvement in both timing accuracy and parameter estimation suggest that decreasing DNAP pausing through these or other methods could be a useful approach to improve the accuracy of molecular recording systems.

### Influence of experimental design on algorithm performance

We observe that errors in tuning parameter estimation in our simulated reaching experiments are not always normally distributed; rather, in a number of neurons, there appear to be several preferred directions that alignments converge upon, including peaks at a neuron’s anti-tuned direction (Fig 3C). This effect persists, although less prominently, when using a non-pausing DNAP (S4 Fig). This is useful to consider given the underlying center-out task in our experiment, where subjects reach in a direction then immediately make a reach back to the center, i.e. the opposite direction of the initial reach. It seemed possible that pathologic alignments could arise from this repetitive temporal structure, where alignments to tuned and anti-tuned templates are effectively identical save for a time-lag. Disrupting this structure through appropriate experimental design could lead to improved accuracy.

**Fig 4:**
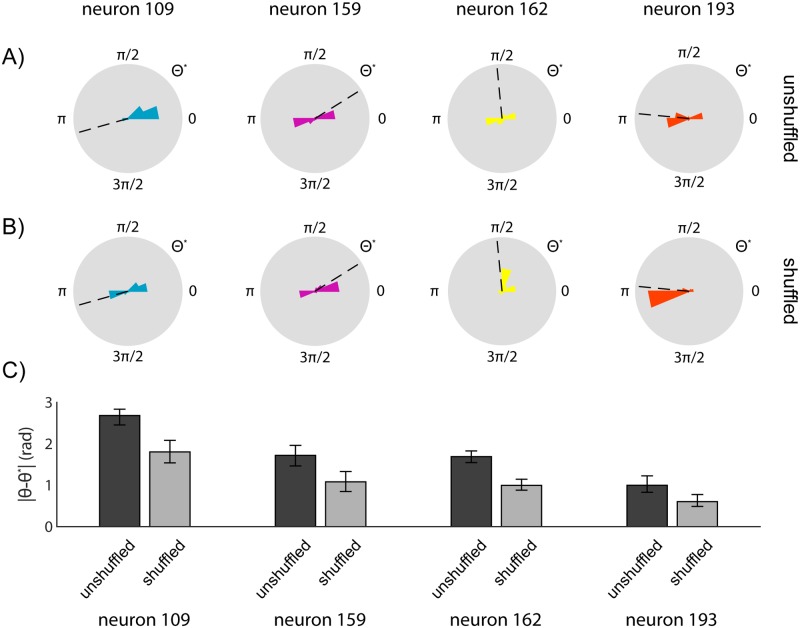
Effects of shuffled dataset on alignment accuracy. Evaluation of synthetic shuffled dataset on alignment performance. Preferred directions were determined using the best alignment to a set of 8 estimates of neural activity. True neural preferred directions were determined using a generalized linear model trained on x- and y-direction hand velocity. **A)** Histograms of algorithm-determined preferred directions of 4 selected neurons using the original dataset. Histograms represent relative frequencies over 100 simulated DNA-based records. Dashed line indicates true neural preferred direction. **B)** Histograms of algorithm-determined preferred directions of 4 selected neurons using a dataset consisting of random 2-second patches of the original dataset. Histograms represent relative frequencies over 100 simulated DNA-based records. Dashed line indicates true neural preferred direction. **C)** Average absolute error in estimating the preferred directions of 4 selected neurons using either the original or shuffled dataset. Error bars represent bootstrapped 95% confidence intervals over 100 trials.

We generated a dataset composed of shuffled 2-second-long patches of neural and kinetics data such that the temporal structure of the original dataset was disrupted. We find that shuffling the data can both reduce selection of anti-tuned preferred directions (Fig 4A and 4B), as well as decrease overall tuning estimation error (Fig 4C). However, it is important to note that the shuffling scheme we describe here does not improve alignment for all neurons, and can even disrupt alignment of neurons that are otherwise predicted correctly (S5 Fig). While this argues against naïve shuffling as a universal strategy, it further demonstrates the effect of an experiment’s temporal structure on alignment accuracy. These findings suggest that experimental design cognizant of alignment-based analysis can improve robustness to pathologic alignments, and thus the feasibility of molecular recording-type experiments.

## Discussion

We describe an algorithm that generates estimates of nucleotide incorporation times for a molecular recording system, along with estimates of parameters that characterize the underlying recorded system. We improve upon *naïve* estimates of these values by incorporating observed experimental data along with a probabilistic description of recorder properties. We apply the algorithm to simulated neuroscience experiments, demonstrating the viability of this algorithm (and the general molecular recording scheme) in a number of scenarios. Our findings suggest that single-strand molecular recording is statistically feasible in neuroscience contexts. Further, by introducing experimental information into our estimation techniques, we improve upon previously-understood limits on the technique. Single-strand recording promises to be a useful technique in neuroscience and biology in general for a number of reasons; establishing a statistical framework for the interpretation of those signals is an important step towards the realization of this technology.

This algorithm is computationally novel, as it incorporates dynamic programming, probabilistic inference, and biologic constraints into a single framework. We modify existing DTW approaches to signal alignment, constraining our action space to physiologically possible actions (e.g. two nucleotides cannot be incorporated at the same time), as well as incorporating beliefs about DNAP kinetics. These constraints have a convenient property in that they restrict our action space to a set that can largely be calculated independently, allowing for parallelization of a dynamic algorithm. While the algorithm maintains the same approximate time complexity of traditional DTW (worst-case of *O*(*NT*)), its inherent parallelism can lead to dramatically decreased runtime.

Further, while not discussed at length here, if recording start or end times are known, variance of incorporation times scale with $\sqrt{N}$ assuming a Poisson-like DNAP. Path-constraint techniques could take advantage of this property, reducing effective worst-case time complexity to $O\left(N^{\frac{1}{2}}T\right)$ and allowing further speed increases [10,28]. These speed improvements are of particular importance due to the inherently large scale of molecular recording: if we want to record from hundreds-of-thousands to millions of neurons, the computational techniques necessary to interpret these signals should scale well.

To this end, there are a number of different biological methods that could be used to explicitly mark the start or end of molecular recorder output, e.g. by delivered analyte pulses or by optogenetic manipulation. These methods could also be used to provide time-coding throughout an experiment, making timing inference substantially easier. Similarly, designing behavioral tasks to modulate neural activity at levels significant enough to be detected, but low enough not to alter behavior, e.g. temporally modulating the brightness of visual stimuli, could be used as an implicit time-coding technique. These experimental methods for encoding timing information into molecular records can work alongside algorithmic alignment methods to improve both timing and parameter inference.

This work also has implications on current work in the biological space. It is useful to understand the effects of DNAPs with different behaviors (e.g. speed, error rate) on the ability to record information, both for our application to molecular recorders, as well as for other approaches that aim to record continuous signals intracellularly. Understanding the general space where recorders work (or fail) is useful not only for determining what kinds of DNAPs we need to find or design, but also for determining which kinds of phenomena might be amenable to study using molecular recorders.

### Biological feasibility and implementation

There are many ways in which existing DNAPs already satisfy the requirements necessary for a single-strand biological recorder, e.g. processivity, speed, calcium-sensitive error rates, and pausing kinetics [19,26,29]. The one property that we have not observed in DNAPs is a calcium-sensitive error rate at physiological concentrations [20]. Further, natural DNAPs tend to be either fast or error-prone, but not generally both; the highest error rates we see in high-incorporation-rate DNAPs are at the low end of what we simulate here [21,30]. In order to develop practical molecular recorders, we will both need to understand how to substantially increase DNAP error rates in processive, high-speed DNAPs, as well as develop a scheme to make DNAP error rates calcium-sensitive at physiologically relevant scales. Alternatively, schemes that do not rely on calcium-tuned error rates, but rather modulate other DNAP properties via calcium, may provide an easier way forward.

### Caveats

#### Need for good predictive models

The success of alignment approaches in this context is dependent on having estimates of neural activity that span the classes of neurons we are interested in recording. That is, we generally have to know the class of phenomenon we are looking for before we are able to look for it. For the recording of more well-characterized brain areas, e.g. V1 or M1, we have at least a general knowledge of the neural response to stimulus or behavior. In these systems, molecular recording would allow for characterization of large populations of neurons based on existing models of neural behavior. Further, ongoing refinement of these models promises to more accurately model neural activity in more areas of the brain, which in turn will increase the applicability of these model-based alignment approaches. For less-well understood tasks however, we run the risk of biasing our recordings toward currently understood neuronal behaviors. While our approach is useful in neuroscience paradigms where we are seeking to classify neurons according to known models or learn their tunings under an assumed model, it does not obviate the need for prior-free exploration of unknown behaviors. This technique will not necessarily allow us to discover unheard-of neural behaviors; rather, it allows us to sense neural activity from neurons we already somewhat understand while greatly increasing the scale at which we study them.

#### Need for tailored experimental design

We have also shown that the success of temporal alignment for molecular recording relies heavily on experimental design. That is, many experimental paradigms may need to be reworked in order to be compatible with this type of analysis, and some may be entirely incompatible with these techniques. Our work provides some general guidelines for experimental design for experiments that utilize molecular recorders. In particular, it suggests that experiments can be manipulated to create unique signatures in their resulting records, given some set of likely neural tunings. As a quality control mechanism, stimulus delivery (or subject activity) should be designed so as not to induce oscillation or other regularities. In addition to designing experiments to avoid pathologic sequences, these experiments could be actively designed to provide unique patterns or time-codes in order to intentionally improve alignment accuracy. Through engineering input data in this way, we can increase the accuracy of this type of alignment algorithm, allowing for more accurate experiments using molecular recorders.

### Implications of work

While many caveats apply to this work, and to the prospect of molecular recorders in general, the results described here are helpful on a number of fronts. On a technical side, we describe a DTW-class algorithm that applies generally to point processes with variable temporal indexing. The algorithm is designed to allow probabilistic interpretation of its output, and can be used to find *maximum a posteriori* alignments to a set of known templates. We provide a highly-parallelized implementation of this algorithm which leverages advances in asynchronous computing techniques. With respect to molecular recorders, we provide a framework for interpretation of recorder output in the face of uncertain recording times. We also provide guidance to the ongoing research that looks to engineer DNAPs for this kind of recording. Perhaps most importantly, we have shown that, should a DNAP with certain properties be developed, we can provide temporal indexing to its output and capture neural behaviors using a molecular recording approach. While this is purely a simulation study, our work sets constraints and goals for the development of DNAPs for massive-scale neural data recording, and outlines experimental scenarios for their successful use.

## Methods

### Algorithm methods

This technique is intended to align a DNA-based recording with no temporal indexing to a longer, time-indexed estimation of calcium activity, a template. It assumes the DNA sequence as a binary “error”/”no error” code, then assesses the similarity of that sequence to a discrete-time continuously-valued estimate of neural activity, the template, via alignment. We use a novel DTW-class algorithm to perform this alignment, incorporating beliefs about DNAP kinetics to limit the space of potential actions.

#### Generative model

We assume some unknown discrete calcium signal, **C** = *c*_1_,…,*c*_*T*_, where *c*_*t*_ ∈ [*c*_*min*_, *c*_*max*_] is the local calcium concentration at some time *t*, and *T* is the number of time-indexed samples included in the recording window. We also have a sequence of correctly- and incorrectly-copied nucleotides, **D** = *d*_1_,…,*d*_*N*_, *d*_*n*_ ∈ {0,1}, where *N* is the number of nucleotides, *d*_*n*_ = 1 denotes a mismatch (error) at position *n*, and *d*_*n*_ = 0 denotes a nucleotide with a correct Watson-Crick basepair.

**Fig 5:**
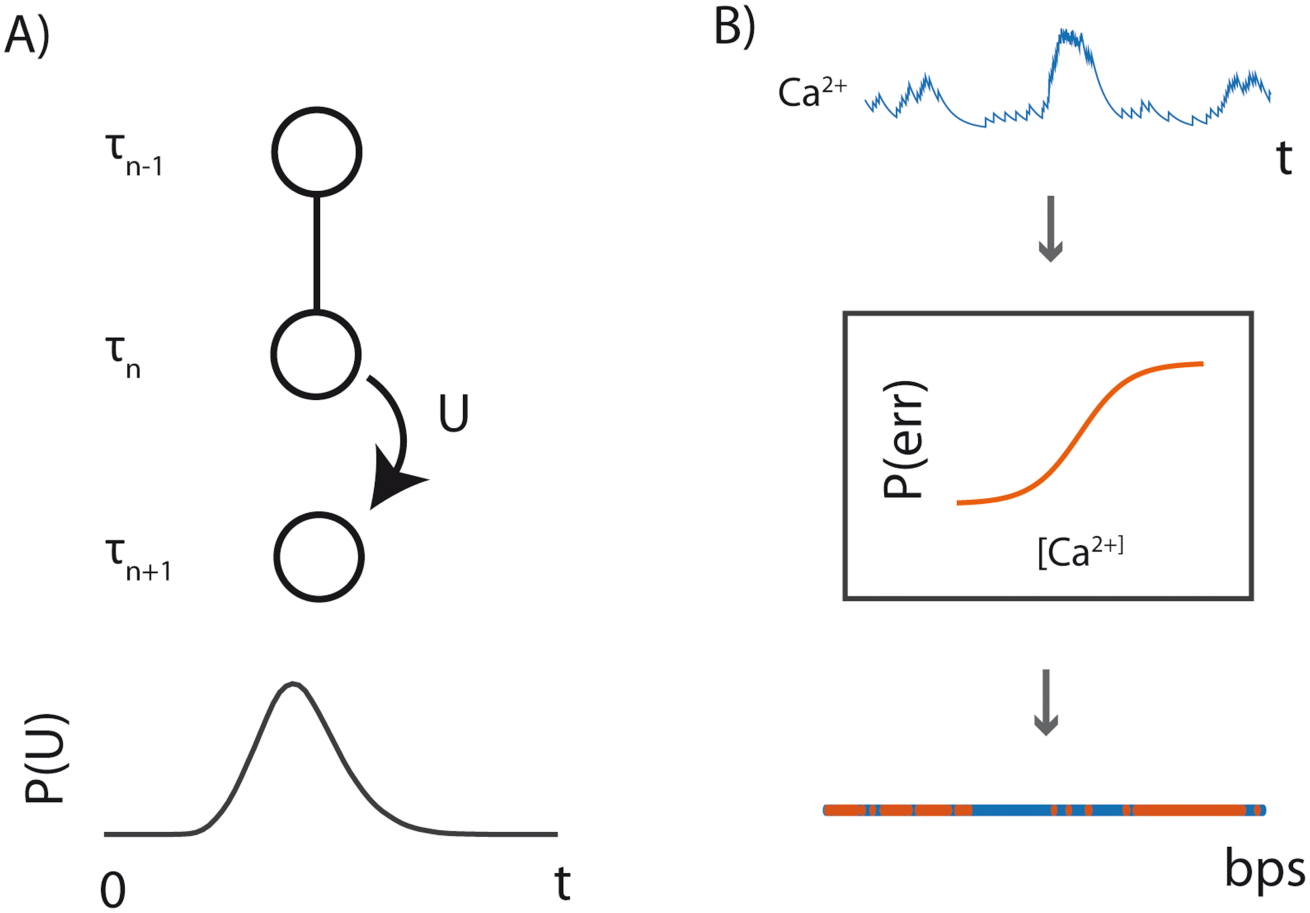
Overview of data generative model. **A)** Stochastic generation of **T**. The incorporation time of a nucleotide, *τ*_*n*_, is defined as *τ*_*n*-1_ + *U* where *U* is a random variable with a distribution that describes the kinetics of the DNAP being used. **B)** Stochastic generation of errors. At each incorporation time *τ*_*n*_, an error is generated with probability $f\left(C_{\tau_{n}}\right)$. Errors in the nucleotide strand are represented by blue regions, correct incorporations are represented by orange regions.

The individual elements of **D** have incorporation times **T** = *τ*_1_,…,*τ*_*N*_ where *τ*_*n*_ ∈ {1,…,*T*} and *τ*_*n*_ < *τ*_*n*+1_ (Fig 5A). We can impose a prior over recording start times *P*(*τ*_1_ = *t*) = *π*_*t*_; we use a uniform prior over an interval $\left[0,\frac{T}{4}\right]$ here to generate data. For 1 < *n* ≤ *N*, *τ*_*n*_ = *τ*_*n*-1_ + *U*, where *U* is drawn from a distribution representative of polymerase kinetics. That is, the distribution of *U* is the distribution of times between nucleotide incorporations. *d*_*n*_ is then drawn from a distribution $P\left(d_{n}=1\right)=f\left(c_{\tau_{n}}\right)$, where *f*(·) is the calcium-dependent error function of the polymerase, and $c_{\tau_{n}}$ is the calcium concentration at incorporation time *τ*_*n*_ (Fig 5B).

Of **C**, **D**, and **T**, we only observe **D**. We wish to infer **C** and **T** using the strand **D** and observed experimental data. To do this, we generate an approximation of **C**, $C^{*}=c_{1}^{*},\ldots ,c_{T}^{*}$, using a model of neural activity that estimates neural calcium response from observed experimental data. We use C* as a template for the alignment of **D**. This alignment allows us to estimate the temporal indexing **T**, which can be used to estimate **C** along with underlying system parameters.

#### Creation of similarity matrix

We generate a similarity matrix **A** between C* and **D** such that $A_{n,t}=\text{ln}P\left(d_{n}|c_{t}^{*},\tau_{n}=t\right)$. That is, **A**_*n*,*t*_ is the log-likelihood of *d*_*n*_ being written at time *t* given the estimated calcium concentration $c_{t}^{*}$ and DNAP error tuning *f*(·). Thus, **A** represents local similarity between each element of **D** and C*.

#### Matrix traversal

After we have generated a local similarity matrix **A**, we then want to find the path T*, an estimate of **T**. To generate this estimate, we find a T* which traverses **A** with maximum likelihood, visiting each *n* ∈ {1,…,*N*} only once, given **A** and the distribution of ***U***. We utilize a dynamic programming approach to estimate the likelihoods of paths through **A**, utilizing the physical requirement *τ*_*n*_ < *τ*_*n*+1_ to constrain our step pattern, i.e. a nucleotide cannot be incorporated earlier in time than its predecessor on the strand, and the Markov assumption *P*(*τ*_*n*_|*τ*_*n*−1_,…,*τ*_1_) = *P*(*τ*_*n*_|*τ*_*n*−1_). This approach, similar to other dynamic time warping algorithms, determines the most-likely path from some starting point to position **A**_*n*,*t*_ by calculating the most-likely paths to some set of penultimate positions **A**_*n*−1,…_ and the accumulated likelihood of those paths, then selecting the path from **A**_*n*−1,…_ to **A**_*n*,*t*_ that gives the highest accumulated likelihood [9,31].

We initialize with log *P*(*τ*_1_ = *t*) = **A**_1,*t*_. At this step, a prior representing knowledge of when reactions likely begin can be incorporated, but is not used here. We then evaluate a likelihood function of some sequence *τ*_1_,…,*τ*_*n*_ that resembles traditional dynamic alignment cost functions such that:

$$\text{ln}P\left(\tau_{n}=t\right)=A_{n,t}+{\text{max}}_{\tau^{\prime }\in t-k,\ldots ,t}\left[\left(1-\omega \right)\text{ln}P\left(\tau_{n-1}=\tau^{\prime }\right)+\omega \cdot \text{ln}P\left(U=t-\tau^{\prime }\right)\right]$$ (1.2)

$$\tau_{n,t}^{\prime }=\text{arg}{\text{max}}_{\tau^{\prime }\in t-k,\ldots ,t}\left[\left(1-\omega \right)\text{ln}P\left(\tau_{n-1}=\tau^{\prime }\right)+\omega \cdot \text{ln}\left(P\left(U=t-\tau^{\prime }\right)\right)\right]$$ (1.3)

where $\tau_{n,t}^{\prime }$ is the most likely time *d*_*n*−1_ was written given *τ*_*n*_ = *t*, *ω* is a parameter that adjusts the relative strength of local similarity, previous similarity, and polymerase kinetics on likelihood, and *k* defines how “far back” we choose to look for the best previous step. Effectively, for any (*n*, *τ*_*n*_), we calculate the most likely $\left(n-1,\tau_{n,\tau_{n}}^{\prime }\right)$. We evaluate *P*(*τ*_*n*_ = *t*) for all pairs (*n*, *τ*_*n*_), *n* ∈ {1,…,*N*} and *τ*_*n*_ ∈ {1,…,*T*}. For each possible (*n*,*t*), we store *P*(*τ*_*n*_ = *t*) and $\tau_{n,t}^{\prime }$.

**Fig 6:**
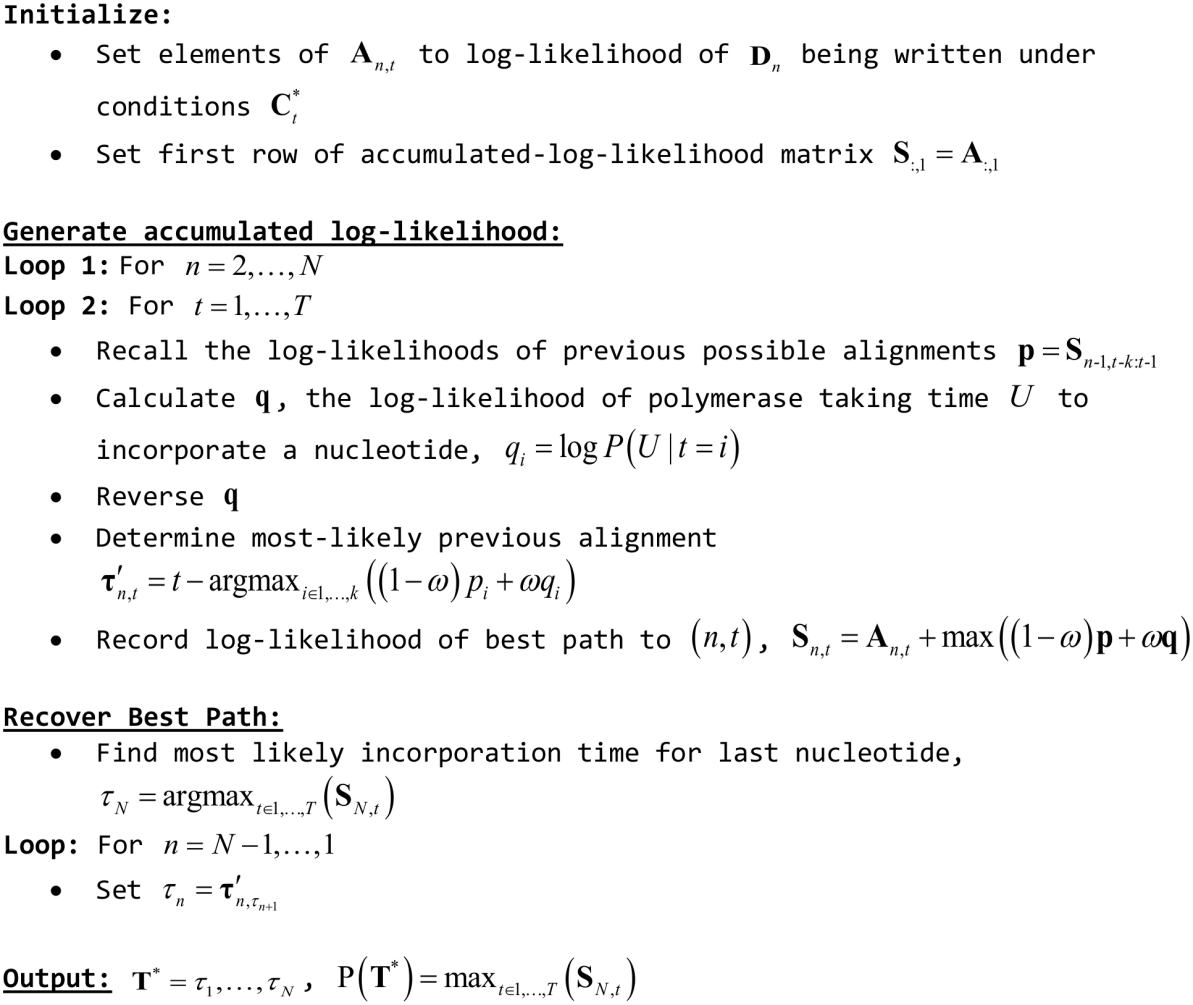
Pseudocode for alignment algorithm.

Once *P*(*τ*_*n*_ = *t*) has been calculated for each (*n*,*t*), we can reconstruct the most likely alignment T*. We find the most likely end point *τ*_*N*_ = argmax_*t*∈0,…,*T*_ *P*(*τ*_*N*_ = *t*), i.e. we select the path T* that ends at the most likely *τ*_*N*_. We then set $\tau_{N-1}=\tau_{N,\tau_{N}}^{\prime }$ and so on for *τ*_*N*−2_,…,*τ*_1_. This algorithm is implemented in pseudocode in Fig 6.

It is useful to note here a structural relationship between our algorithm and a DTW step-pattern variant proposed by Itakura [32,33]. Both algorithms only use data from **A**_*n*−1,1…*T*_ to calculate **A**_*n*,*t*_, which implies that the calculations for any two elements in a row are independent; we extend the Itakura action space and remove several other restrictions from potential paths. The Itakura step pattern is intended as a path-bounding scheme; while we do not implement bounding explicitly here, it is performed implicitly with our choice of *k*. Thus, the algorithm as described is an approximation of the true *maximum a posteriori* solution, as we do not evaluate the entire solution space. We also inherit several attractive attributes with respect to parallelism from Itakura, which we discuss later.

#### Parallelization of alignment algorithm

We have described an algorithm with worst-case time complexity *O*(*NTk*) and *k* threads that can be operated on concurrently, i.e. all operations in the vector addition (1 − *ω*)**p** + *ω***q** can be performed independently. In comparison, traditional DTW is worst-case time complexity *O*(*NT*) for our purposes, and has 3 threads that can be operated on concurrently. To calculate an element **S**_*n*,*t*_ using our algorithm, we only require values from row *n* − 1, indicating that the computations for **S**_*n*,*t*_, *t* ∊ 1,…, *T* are independent. It follows that that we actually have *Tk* threads that can be operated on concurrently, i.e. the operation (1 − *ω*)**p** + *ω***q** for each **S**_*n*,*t*_, rather than k. To implement this, we pre-generate **q** and **p** for each *t* ∊ 1,…, *T*; the algorithm can then be carried out for each *t* concurrently for a given *n*. While algorithm complexity does not change, we improve runtime by a factor of up to *T* via parallelization.

#### Maximum-likelihood template selection

In order to generate an accurate estimate C*, we need to know how a neuron is tuned to its inputs. As we do not know this *a priori*, we instead generate multiple candidates $C_{m}^{†}$ from some set of possible neural tunings and let C* be the estimate $C_{m}^{†}$ with the most-likely alignment to the data **D**. There are time-indexed experimental variables **X** = **x**_**1**_, …, **x**_**T**_, and a set of tunings Θ = {*g*_1_(·), …, *g*_*M*_(·)}, where $C_{m}^{†}=g_{m}\left(X\right)$. Simply, Θ enumerates the possible ways we believe a neuron transforms experimental covariates (e.g. movement, delivered stimuli) into activity. We now select the tuning *g*_*m*_(·) that provides the most likely alignment to our observed data **D**. We do this by aligning the observed **D** with each $C_{m}^{†}$, selecting $C^{*}=\text{arg}{\text{max}}_{C^{†}\in \{g_{1}(X),\ldots ,g_{M}(X)\}}P\left({Τ}^{*}|D,C^{†}\right)$ , the intuition being that **D** should most closely resemble the signal that generated it. Once we have selected a most-likely tuning from the ensemble Θ and alignment T*, we then estimate actual neural tuning directly from aligned DNA.

#### Approximations

We significantly reduce the computational requirements of the algorithm by using downsampled approximations of **D** and $C_{m}^{†}$. To abstract our data, we first decimate **C**^**†**^, effectively taking a binned average with bin size *L*_*C*_. We then bin **D** into bins of size *L*_*D*_, letting $d_{n\prime }=\sum_{n=Di}^{D(i+1)}d_{n}$ be the total number of errors in bin *n*′.

We then align the downsampled $C_{m}^{†}$ and **D** using the algorithm described above, altering the cost-function for **A**_*n*,*t*_:

$$A_{n\prime ,t\prime }=\text{log}P\left(d_{n\prime }|c_{m,t\prime }^{†},\tau_{n\prime }=t\prime \right)$$ (1.4)

where $d_{n\prime }\;\sim \text{Binomial}\left(L_{D},f_{e}\left(c_{m,t\prime }^{†}\right)\right)$. Through this, we generate an approximate most-likely alignment ${\text{T}}_{a}^{\prime }$. **T**_**a**_ is a low-resolution alignment; we recover a full-nucleotide alignment **T**′ by interpolating between points on ${\text{T}}_{a}^{*}$.

### Experimental methods

#### DNAP parameter evaluation

We generated an initial stimulation trace **I** by concatenating 400 periods of stimulation, length 5s with intensity *I*_*e*_ ~ Uniform(0, 1). We then simulate neural firing rate **λ**, *λ*_*t*_ = *mI*_*t*_ + *λ*_*min*_, with *m* = 0.05 spikes · ms^−1^ · unit of stim^−1^ and *λ*_*min*_ = 0 spikes · ms^−1^, and generated spiking activity *s*_*t*_ ~ Bernoulli(*λ*_*t*_). We then generate a calcium trace **C** by convolving spikes with an exponential filter with decay *τ* = 200 ms. We can then calculate the effective relationship **C** ∝ *m*_*ca*_**I**. We also generate an accurate estimate of calcium, C*, by convolving **λ** with the same exponential filter.

DNAP kinetic parameters were chosen to reflect DNAP extension and pausing behavior used to generate the data. These parameters, other than calcium response, are generally reflective of known DNAPs [19,34]. We generate a DNA-based record **D** from **C** as above, using the “Base Parameter Evaluation DNAP” in Table 1. We then align **D** to C* using the algorithm parameters for “Parameter Evaluation Experiments” in Table 2. Timing accuracy for each alignment is evaluated as above. Slope accuracy is evaluated by first calculating the error-tuning curve over the range of **C**, transforming the error-tuning curve with *f*^−1^(·), then calculating the slope of the resulting calcium-tuning curve, $m_{ca}^{*}$. We report the ratio $\frac{m_{ca}^{*}}{m_{ca}}$. 95% confidence intervals were generated by bootstrapping over alignment results for 50 DNA strands at each reported point.

**Table 1:** DNAP simulation parameters.

		Center-Out DNAP	“Optimized” Center-Out DNAP	Base Parameter Evaluation DNAP
Error function and parameters	*f*(·)	$R_{max}\cdot \frac{1}{1+\text{exp}\left[b\left(C-C_{0}\right)\right]}$	$R_{max}\cdot \frac{1}{1+\text{exp}\left[b\left(C-C_{0}\right)\right]}$	$R_{max}\cdot \frac{1}{1+\text{exp}\left[b\left(C-C_{0}\right)\right]}$
	*R*_*max*_	0.5	0.5	0.5
	b	1	1	1
	*C*_0_	0	0	0
Kinetic distribution and parameters	U (Distribution)	pause· Exp(*λ*_*p*_) + (1 − pause) Gamma(*α*, *β*)	Gamma(*α*, *β*)	pause· Exp(*λ*_*p*_) + (1 − pause) Gamma(*α*, *β*)
	Pause (Distribution)	Binomial(*p*_*pause*_)	N/A	Binomial(*p*_*pause*_)
	*λ*_*p*_	2s	N/A	2s
	α	1	1	1
	β	10ms	10ms	10ms
	*p*_*pause*_	0.001	N/A	0.01
	# of basepairs	12,000	12,000	10,000

**Table 2:** Default alignment parameters.

	*k* (ms)	*ω*	Ca^2+^ downsample rate (ms/sample)	DNA downsample rate (nt/sample)
Parameter Evaluation Experiments	2000	$\frac{1}{100}$	50	100
Center-Out Experiments	2000	$\frac{1}{240}$	50	25

#### Center-Out reaching experiments

We obtained kinetic and neural activity records from Flint 2012 via the DREAM database, using data from Subject 1 [25]. We preprocess the data by concatenating all 194 trials, discarding data where hand velocity either exceeded 0.4 m/s or fell below 0.05 m/s, and truncating traces to 260 seconds. We generated a calcium trace **C** by convolving spikes with an exponential filter with decay *τ* = 200 ms. To generate DNA-based signals, we first determine incorporation times **T** by drawing nucleotide incorporation times from distribution *U* as described in Table 1, using either the “Center-Out” or “Optimized Center-Out” parameters. We then determine whether each nucleotide was a correct or incorrect incorporation as $d_{n}\;\sim \text{Bernoulli}\left(f\left(C_{\tau_{n}}\right)\right)$, using the *f*(·) described in Table 1.

We select 8 candidate preferred directions, evenly spaced on [0, 2*π*], as the parametrization Θ for our estimates of neural activity. We calculate expected firing rates $\lambda_{m}^{\prime }$ for each of these candidate preferred directions, using recorded hand velocities and a cosine-tuning model, setting minimum and maximum firing rates to values representative of the recorded population; we set *λ*_*min*_ = 10 spikes · s^−1^ and *λ*_*max*_ = 150 spikes · s^−1^, which generally represents the observed neural population. We convolve $\lambda_{m}^{\prime }$ with an exponential kernel described above to generate estimated calcium $C_{m}^{†}$.

The generated DNA strand **D** is aligned to each estimated calcium trace $C_{m}^{†}$, using algorithm parameters (ω, k, and downsample rates) as described in Table 2. The most likely alignment from these is selected for analysis. We calculate RMSD for a given alignment as $\frac{1}{N}{\left[\sum_{n=1}^{N}{\left({Τ}_{n}-{Τ}_{n}^{*}\right)}^{2}\right]}^{1/2}$. We evaluate the preferred reach direction of the neuron directly from neural data using standard generalized linear modeling techniques, fitting x- and y-components of reach velocity to 1ms-binned spike counts. We use this direct preferred direction as a ground truth for evaluating algorithm performance. For the aligned DNA-based records, we evaluate the estimated preferred direction of the neuron using a generalized linear model, fitting reach instantaneous velocity to error counts at each nucleotide incorporation time.

For each analyzed neuron, we generate 100 DNA-based records, align each record to each estimated calcium trace, and evaluate timing and parameter estimates using the maximum-likelihood alignment for each record. Confidence intervals for error estimates are generated by bootstrap.

For all-neuron analysis, 100 strands were generated and aligned as above. Neurons were filtered based on average firing rate > 20 spikes/s and a McFadden’s pseudo-R^2^ > 0.05, calculated for a Poisson generalized linear model fitting x- and y- hand velocities to spike rate.

#### Timing data

For each trial, a strand of given length is aligned to a 2,000s calcium trace without downsampling. The total time elapsed for each alignment was recorded. 10 trials were performed for each data point, averages are presented. Algorithms were implemented in MATLAB (MathWorks Inc.), and evaluated on an Intel i7-3520M 2.9GHz CPU and an NVidia NVS 5200M discrete graphics card.

## Supporting information

S1 Fig Relative algorithm performance. **A)** Schematic of considered algorithms. Dark purple elements indicate the current element being calculated, light purple elements are elements still to be computed. Grey elements represent previously computed results needed to evaluate the current element. Traditional DTW consists of element-wise computation of an accumulated cost function, iterated over both dimensions of the cost matrix. The looped version of our algorithm implements element-wise computation of our modified accumulated cost function, also iterated over both dimensions of the cost matrix. Our vectorized algorithm calculates the accumulated cost functions of all elements along a given dimension, and iterates over the other dimension. **B)** Computational speed of the GPU-implemented algorithm relative to other implementations. We compare to a looped implementation of our algorithm, an implementation of traditional DTW, and our optimized algorithm using on a single CPU core. We evaluate computation time during the alignment of a single DNA input of a given length to a constant-length (2,000 second) template; values plotted represent average over ten trials. Dashed line indicates GPU-implemented performance.
(DOCX)

S2 Fig Using optimal templates for alignment. Timing and neural parameter estimation when using either the best alignment from a set of 8 templates generated from potential neural preferred directions on [0,2π] (blue), or from a template generated using the true neural preferred direction (orange). Results are shown for each of the three individual neurons analyzed in the main text. Histograms represent distribution over 100 trials. **A)** Distribution of timing errors for DNA-based records when aligned to the indicated template. B) Distribution of estimated neural preferred directions when aligned to the indicated template. Dashed lines indicate the true neural preferred direction, estimated from neural data.
(DOCX)

S3 Fig Alignment accuracy over a neural population. Cumulative fractions of the neural population that have alignment statistics at or below a given cutoff. Traces are provided for both the entire dataset (blue) and a subset of neurons with average firing rate greater than 20 spikes/s and a model McFadden’s pseudo-R^2^ > 0.05 (purple). **A)** Proportion of population with average trial RMSD less than indicated value. **B)** Proportion of population with median trial RMSD less than indicated value. **C)** Proportion of population with absolute error in estimated preferred direction |*θ* − θ*| less than indicated value.
(DOCX)

S4 Fig Plausible vs. optimal DNAPs in alignment. Timing and neural parameter estimation when using “natural” (blue) or “optimized” (orange) pausing DNAPs (see Methods). Results are shown for each of the three individual neurons analyzed in the main text. DNA-based records were generated using the indicated DNAP and aligned to a set of 8 templates generated from potential neural preferred directions on [0,2π]; most-likely alignments were used to generate timing and tuning error. Histograms represent distribution of values over 100 trials. **A)** Distribution of timing errors for DNA-based records generated using the indicated DNAP. **B)** Distribution of estimated neural preferred direction for DNA-based records generated using the indicated DNAP. Dashed lines indicate the true neural preferred direction, estimated directly from neural data.
(DOCX)

S5 Fig Shuffled datasets offer heterogeneous effects for alignment accuracy. Evaluation of synthetic shuffled dataset on alignment performance for a set of neurons that do not exhibit improvement using a shuffled dataset. Preferred directions were determined using the best alignment to a set of 8 estimates of neural activity. True neural preferred directions were determined using a generalized linear model trained on x- and y-direction hand velocity. **A)** Histograms of algorithm-determined preferred directions of 5 selected neurons using the original dataset. Histograms represent relative frequencies over 100 simulated DNA-based records. Dashed line indicates true neural preferred direction. **B)** Histograms of algorithm-determined preferred directions of 5 selected neurons using a dataset consisting of random 2-second patches of the original dataset. Histograms represent relative frequencies over 100 simulated DNA-based records. Dashed line indicates true neural preferred direction. **C)** Absolute error in estimating the preferred directions of 5 selected neurons using either the original or shuffled dataset. Error bars represent bootstrapped 95% confidence intervals.
(DOCX)
