# Supplementary material for: Nucleotide-time alignment for molecular recorders
Source: PLoS Comput Biol. 2017 May 1;13(5):e1005483. doi: 10.1371/journal.pcbi.1005483 (PMC5432193; doi:10.1371/journal.pcbi.1005483)
Supplement: S1 Fig — A) Schematic of considered algorithms. Dark purple elements indicate the current element being calculated, light purple elements are elements still to be computed. Grey elements represent previously computed results needed to evaluate the current element. Traditional DTW consists of element-wise computation of an accumulated cost function, iterated over both dimensions of the cost matrix. The looped version of our algorithm implements element-wise computation of our modified accumulated cost function, also iterated over both dimensions of the cost matrix. Our vectorized algorithm calculates the accumulated cost functions of all elements along a given dimension, and iterates over the other dimension. B) Computational speed of the GPU-implemented algorithm relative to other implementations. We compare to a looped implementation of our algorithm, an implementation of traditional DTW, and our optimized algorithm using on a single CPU core. We evaluate computation time during the alignment of a single DNA input of a given length to a constant-length (2,000 second) template; values plotted represent average over ten trials. Dashed line indicates GPU-implemented performance. (DOCX) [file pcbi.1005483.s001.docx]

| 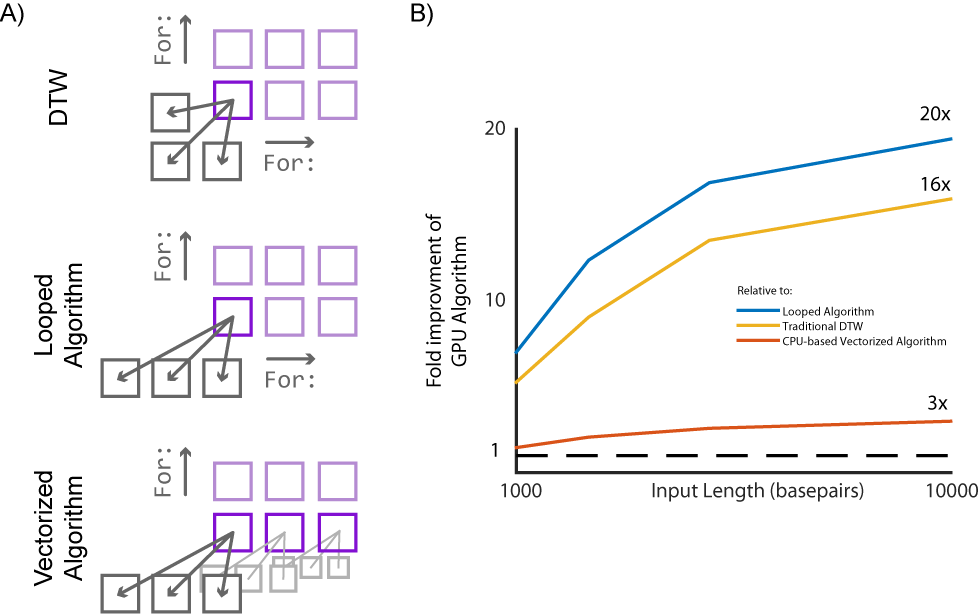 |
| --- |
| **Supplemental Figure 1: Relative Algorithm Performance**  **A)** Schematic of considered algorithms. Dark purple elements indicate the current element being calculated, light purple elements are elements still to be computed. Grey elements represent previously computed results needed to evaluate the current element. Traditional DTW consists of element-wise computation of an accumulated cost function, iterated over both dimensions of the cost matrix. The looped version of our algorithm implements element-wise computation of our modified accumulated cost function, also iterated over both dimensions of the cost matrix. Our vectorized algorithm calculates the accumulated cost functions of all elements along a given dimension, and iterates over the other dimension. **B)** Computational speed of the GPU-implemented algorithm relative to other implementations. We compare to a looped implementation of our algorithm, an implementation of traditional DTW, and our optimized algorithm using on a single CPU core. We evaluate computation time during the alignment of a single DNA input of a given length to a constant-length (2,000 second) template; values plotted represent average over ten trials. Dashed line indicates GPU-implemented performance. |
