# Supplementary material for: Nucleotide-time alignment for molecular recorders
Source: PLoS Comput Biol. 2017 May 1;13(5):e1005483. doi: 10.1371/journal.pcbi.1005483 (PMC5432193; doi:10.1371/journal.pcbi.1005483)
Supplement: S2 Fig — Timing and neural parameter estimation when using either the best alignment from a set of 8 templates generated from potential neural preferred directions on [0,2π] (blue), or from a template generated using the true neural preferred direction (orange). Results are shown for each of the three individual neurons analyzed in the main text. Histograms represent distribution over 100 trials. A) Distribution of timing errors for DNA-based records when aligned to the indicated template. B) Distribution of estimated neural preferred directions when aligned to the indicated template. Dashed lines indicate the true neural preferred direction, estimated from neural data. (DOCX) [file pcbi.1005483.s002.docx]

| 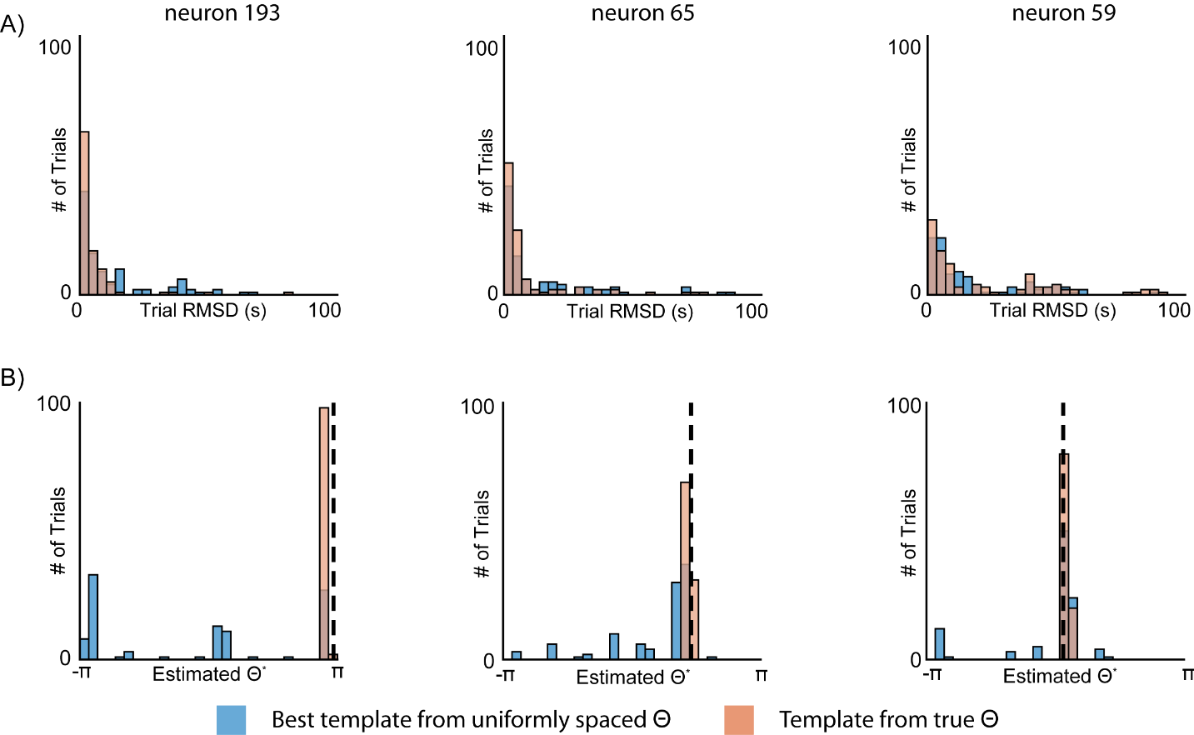 |
| --- |
| **Supplemental Figure 2: Using Optimal Templates for Alignment**  Timing and neural parameter estimation when using either the best alignment from a set of 8 templates generated from potential neural preferred directions on [0,2π] (blue), or from a template generated using the true neural preferred direction (orange). Results are shown for each of the three individual neurons analyzed in the main text. Histograms represent distribution over 100 trials. **A)** Distribution of timing errors for DNA-based records when aligned to the indicated template. B**)** Distribution of estimated neural preferred directions when aligned to the indicated template. Dashed lines indicate the true neural preferred direction, estimated from neural data. |
