# Supplementary material for: Nucleotide-time alignment for molecular recorders
Source: PLoS Comput Biol. 2017 May 1;13(5):e1005483. doi: 10.1371/journal.pcbi.1005483 (PMC5432193; doi:10.1371/journal.pcbi.1005483)
Supplement: S3 Fig — Cumulative fractions of the neural population that have alignment statistics at or below a given cutoff. Traces are provided for both the entire dataset (blue) and a subset of neurons with average firing rate greater than 20 spikes/s and a model McFadden’s pseudo-R2 > 0.05 (purple). A) Proportion of population with average trial RMSD less than indicated value. B) Proportion of population with median trial RMSD less than indicated value. C) Proportion of population with absolute error in estimated preferred direction |θ − θ*| less than indicated value. (DOCX) [file pcbi.1005483.s003.docx]

| 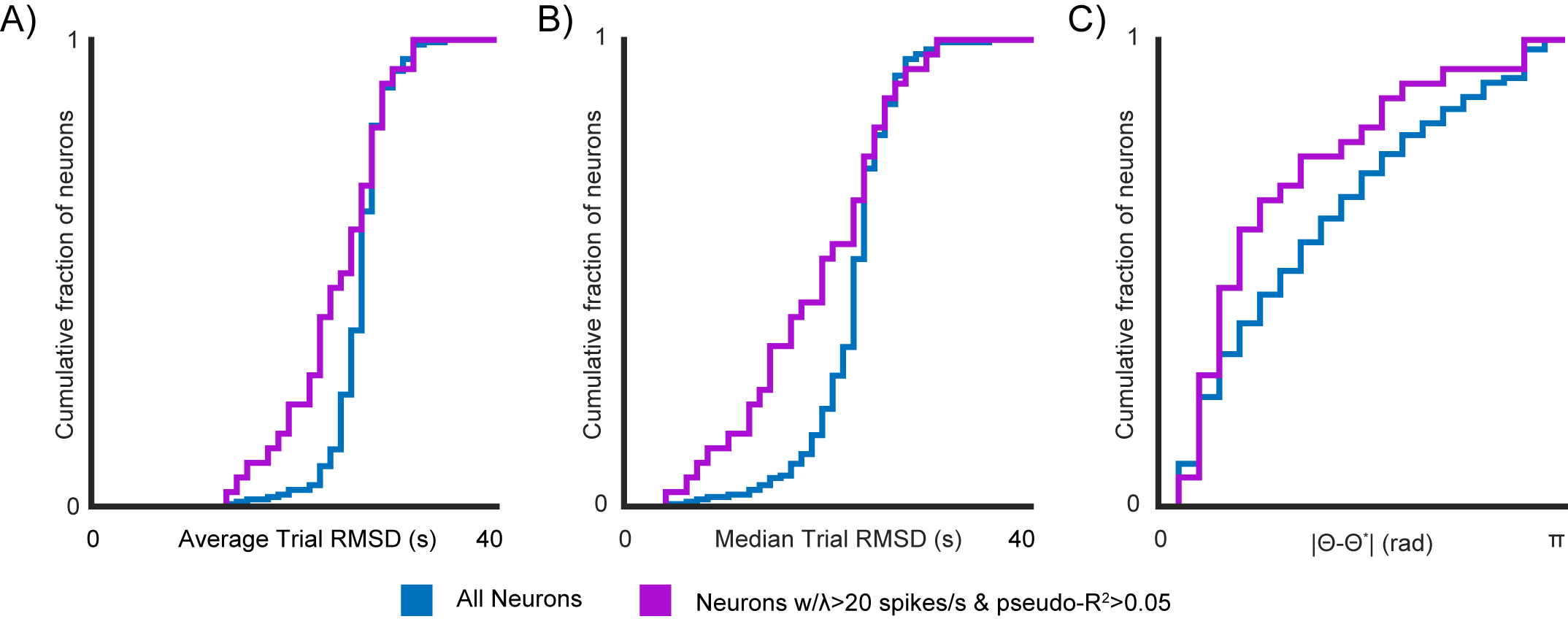 |
| --- |
| **Supplemental Figure 3: Alignment accuracy over a neural population**  Cumulative fractions of the neural population that have alignment statistics at or below a given cutoff. Traces are provided for both the entire dataset (blue) and a subset of neurons with average firing rate greater than 20 spikes/s and a model McFadden’s pseudo-R^2^ > 0.05 (purple). **A)** Proportion of population with average trial RMSD less than indicated value. **B)** Proportion of population with median trial RMSD less than indicated value. **C)** Proportion of population with absolute error in estimated preferred direction $\vert\theta-\theta^{*}\vert$ less than indicated value. |
