# Supplementary material for: Nucleotide-time alignment for molecular recorders
Source: PLoS Comput Biol. 2017 May 1;13(5):e1005483. doi: 10.1371/journal.pcbi.1005483 (PMC5432193; doi:10.1371/journal.pcbi.1005483)
Supplement: S4 Fig — Timing and neural parameter estimation when using “natural” (blue) or “optimized” (orange) pausing DNAPs (see Methods). Results are shown for each of the three individual neurons analyzed in the main text. DNA-based records were generated using the indicated DNAP and aligned to a set of 8 templates generated from potential neural preferred directions on [0,2π]; most-likely alignments were used to generate timing and tuning error. Histograms represent distribution of values over 100 trials. A) Distribution of timing errors for DNA-based records generated using the indicated DNAP. B) Distribution of estimated neural preferred direction for DNA-based records generated using the indicated DNAP. Dashed lines indicate the true neural preferred direction, estimated directly from neural data. (DOCX) [file pcbi.1005483.s004.docx]

| 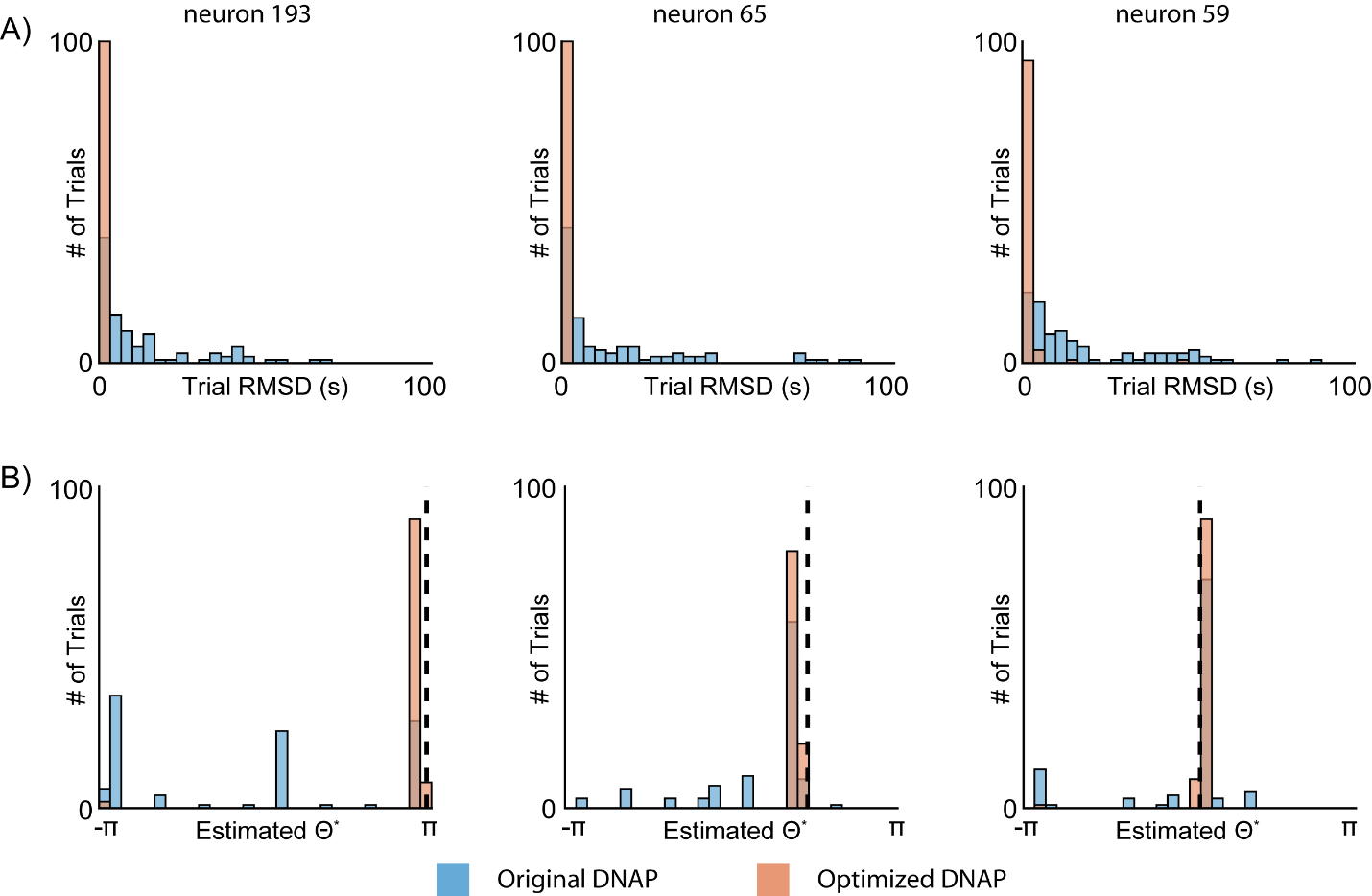 |
| --- |
| **Supplemental Figure 4: Plausible vs. Optimal DNAPs in Alignment**  Timing and neural parameter estimation when using “natural” (blue) or “optimized” (orange) pausing DNAPs (see Methods). Results are shown for each of the three individual neurons analyzed in the main text. DNA-based records were generated using the indicated DNAP and aligned to a set of 8 templates generated from potential neural preferred directions on [0,2π]; most-likely alignments were used to generate timing and tuning error. Histograms represent distribution of values over 100 trials. **A)** Distribution of timing errors for DNA-based records generated using the indicated DNAP. **B)** Distribution of estimated neural preferred direction for DNA-based records generated using the indicated DNAP. Dashed lines indicate the true neural preferred direction, estimated directly from neural data. |
