# Supplementary material for: Nucleotide-time alignment for molecular recorders
Source: PLoS Comput Biol. 2017 May 1;13(5):e1005483. doi: 10.1371/journal.pcbi.1005483 (PMC5432193; doi:10.1371/journal.pcbi.1005483)
Supplement: S5 Fig — Evaluation of synthetic shuffled dataset on alignment performance for a set of neurons that do not exhibit improvement using a shuffled dataset. Preferred directions were determined using the best alignment to a set of 8 estimates of neural activity. True neural preferred directions were determined using a generalized linear model trained on x- and y-direction hand velocity. A) Histograms of algorithm-determined preferred directions of 5 selected neurons using the original dataset. Histograms represent relative frequencies over 100 simulated DNA-based records. Dashed line indicates true neural preferred direction. B) Histograms of algorithm-determined preferred directions of 5 selected neurons using a dataset consisting of random 2-second patches of the original dataset. Histograms represent relative frequencies over 100 simulated DNA-based records. Dashed line indicates true neural preferred direction. C) Absolute error in estimating the preferred directions of 5 selected neurons using either the original or shuffled dataset. Error bars represent bootstrapped 95% confidence intervals. (DOCX) [file pcbi.1005483.s005.docx]

| 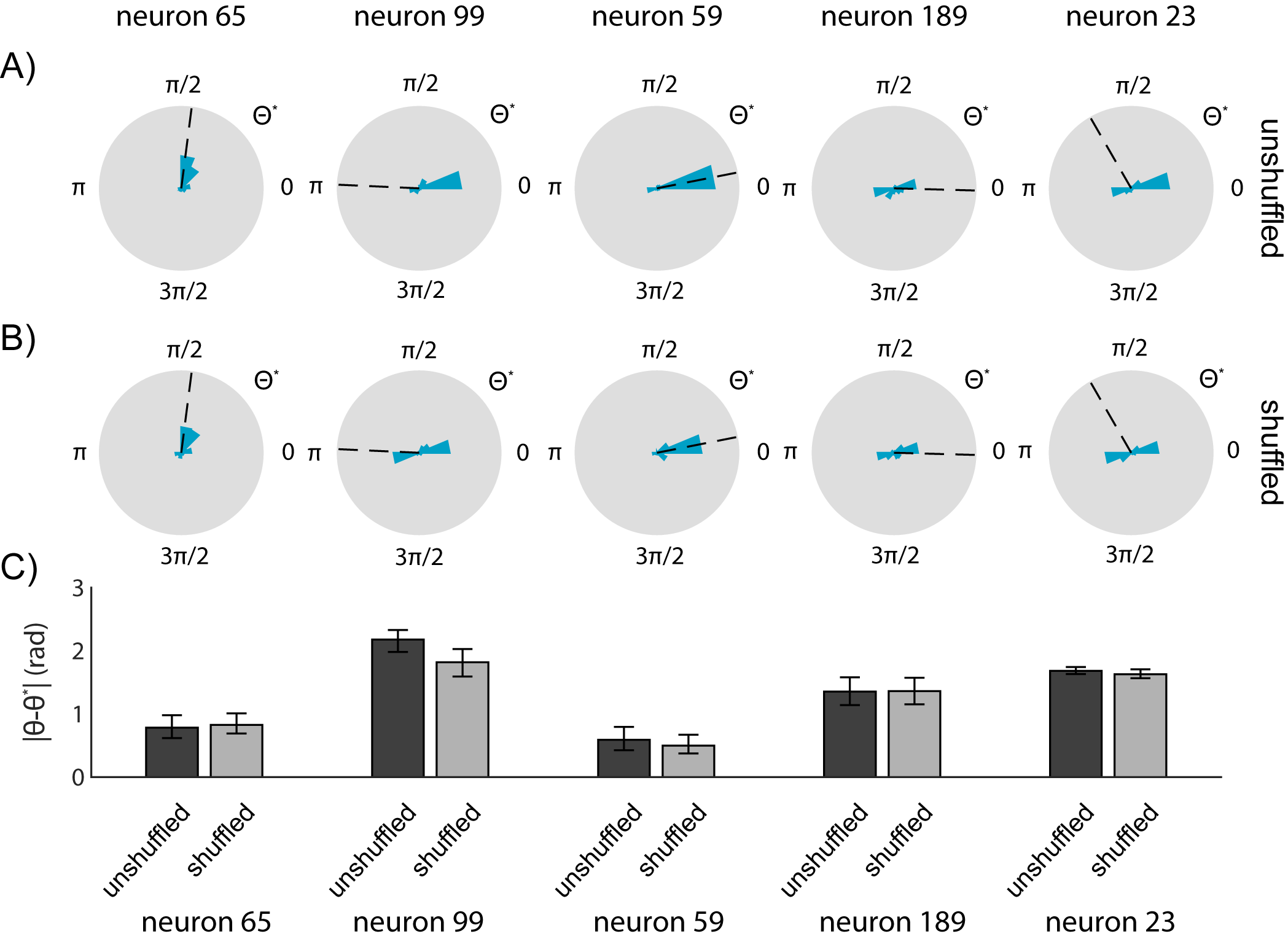 |
| --- |
| **Supplemental Figure 5: Shuffled datasets offer heterogeneous effects for alignment accuracy**  Evaluation of synthetic shuffled dataset on alignment performance for a set of neurons that do not exhibit improvement using a shuffled dataset. Preferred directions were determined using the best alignment to a set of 8 estimates of neural activity. True neural preferred directions were determined using a generalized linear model trained on x- and y-direction hand velocity. **A)** Histograms of algorithm-determined preferred directions of 5 selected neurons using the original dataset. Histograms represent relative frequencies over 100 simulated DNA-based records. Dashed line indicates true neural preferred direction. **B)** Histograms of algorithm-determined preferred directions of 5 selected neurons using a dataset consisting of random 2-second patches of the original dataset. Histograms represent relative frequencies over 100 simulated DNA-based records. Dashed line indicates true neural preferred direction. **C)** Absolute error in estimating the preferred directions of 5 selected neurons using either the original or shuffled dataset. Error bars represent bootstrapped 95% confidence intervals. |
